# CENPA regulates tumor stemness in lung adenocarcinoma

**DOI:** 10.18632/aging.204167

**Published:** 2022-07-11

**Authors:** Qi-Ying Yu, Hui Liu, Chen Liu, Yuan Xiang, Qi-Bei Zong, Jun Wang, Hui-Min Zhang, Cheng-Chen Xu, Jia-Peng Li, Xing-Hua Liao

**Affiliations:** 1 Institute of Biology and Medicine, College of Life and Health Sciences, Wuhan University of Science and Technology, Hubei 430081, P.R. China; 2 Department of Medical Laboratory, Central Hospital of Wuhan, Tong Ji Medical College, Hua Zhong University of Science and Technology, Hubei 430014, P.R. China

**Keywords:** CENPA, cancer stem cell, lung adenocarcinoma

## Abstract

Lung adenocarcinoma is a malignant and fatal respiratory disease. However, due to its complex pathogenesis and poorly effective therapeutic options, accurate early diagnosis and prognosis remain elusive. Now, there is increasing evidence that tumor stem cells are involved in tumorigenesis, metastasis, relapse, resistance to chemotherapy and radiotherapy and are one of the reasons why tumors cannot be cured. The mRNA expression based-stemness index (mRNAsi) is a parameter obtained by Malta and his colleagues applying innovative one-class logistic regression machine learning algorithm (OCLR) on mRNA expression in normal stem cells and their progeny. It is a valid evaluation parameter and is currently employed to evaluate the degree of differentiation of a certain tumor. In this study, we first used WGCNA and the software Cytoscape to obtain key modules and hub genes. We then applied LASSO regression analysis to calculate the genes in the key module to obtain a six-gene risk model. Moreover, the accuracy of this model was validated. Finally, we took the intersection of hub genes and risk genes and validated CENPA as both a tumor stemness regulator and a tumor prognostic factor in lung cancer.

## INTRODUCTION

Lung cancer possesses a high mortality rate, with lung adenocarcinoma accounting for approximately 50% of all types of lung cancer [1, 2]. Because it is difficult to diagnose the disease in its early stages, most patients are diagnosed at a malignant stage, which leaves patients without access to surgery to treat their cancer. Despite significant improvements in treatment techniques such as chemotherapy, molecular targeted Therapy and immunotherapy, the 5-year survival rate for patients after diagnosis remains below 15% [3]. There is an actual demand to identify a new therapeutic and prognostic target to improve the understanding of tumor pathogenesis and treatment.

Bioinformatics-based disease risk analysis has become an important and routine strategy for disease diagnosis, treatment and prognosis [4]. Weighted gene co-expression network analysis (WGCNA) is an effective bioinformatic analysis method often used to obtain modules with highly correlated gene expression patterns and the relationship between they and their clinical characteristics in large numbers of samples [5]. Unlike the focus on differentially expressed genes, the WGCNA algorithm defines the gene co-expression correlation matrix, and constructs hierarchical clustering trees accordingly [6]. By using WGCNA, we can search for key genes in the module of interest and after validation they can be used as targets for subsequent treatment, diagnosis or prognosis.

With the development of RNA sequencing or single-cell sequencing technology, the heterogeneity of tumor is becoming well known and the study of sequencing data has shown that tumor tissues are organized into diverse populations of cells. Among these cell subpopulations, one of them is named tumor stem cells. As the name suggests, tumor stem cells possess some properties of regular stem cells. They can renew themselves by division and have the ability to differentiate into other tumor cell types. Currently, it is widely accepted that resistances to existing conventional therapies are one of their characteristics, and that they act as an indispensable role in the tumorigenesis, progression and metastasis in tumors [7–9].

In this work, firstly, according to both TCGA lung adenocarcinoma (LUAD) cohorts and cancer stemness indices, we obtained the most significant cancer stemness index module through the implementation of the WGCNA calculation and hub genes [10]. Secondly, we then applied LASSO and cox regression to the genes in the above module to construct a prognostic model. This model contains six genes, they are CCNB1 CCNA2 CENPA TTK NEK2 PRC1. Thirdly, we analyzed the intersection of prognostic model genes and hub genes and finally identified, Centromere Protein A, CENPA as the subject of the study. We also conducted a series of experiments to investigate the effects of CENPA on stemness maintenance and cell proliferation. Our results support that CENPA is a useful prognostic biomarker and tumor stemness regulator to help in LUAD prediction, disease management and therapy.

## MATERIALS AND METHODS

### Colony formation assay

LUAD cell line A549 were resuspended to 1×10^3^ cells/mL and seeded in 6-well plates. After two weeks of incubation, the cells were fixed with 4% paraformaldehyde and stained with 0.1% crystal violet. The number of colonies was calculated after three times of PBS washing.

### CCK-8 assay

The CCK-8 assay (Dojindo, Japan) was utilized to test the proliferation of the cells. 10,000 cells were seed in ninety-six well plates. The assay was carried out according to the instructions and the absorbance was measured at 450 nm with an enzyme marker at 0 H, 24 H, 48 H and 72 H after inoculation respectively.

### 5-Ethynyl-2′-deoxyuridine (EdU) assay

Cells were inoculated on 14 mm cell coverslip and cultured overnight. The medium was removed and incubated with medium containing 20 mM EDU (Thermo Fisher, USA) for two hours in a 37°C incubator. Follow the instructions for the subsequent procedure. Olympus FV3000 confocal camera was used to photograph EDU-positive cells.

### Immunofluorescence and confocal imaging

Immunofluorescence of CD44 and EpCAM was used to detect the protein expression. Briefly, cells were inoculated on coverslips and cultured overnight. The medium was removed and subsequently washed cell three times with PBS and then fixed at room temperature in 4% paraformaldehyde followed by 0.5% triton-x100 permeabilization. 1% BSA was used to block the non-specific antigen for 1 hour and the Anti-CD44 (ABclonal, China), Anti-EpCAM (ABclonal, China) antibodies were added and incubated 12 h at 4°C. Cy3-conjugated anti-rabbit (Abcam, USA) and FITC-conjugated anti-rabbit (Abcam, USA) secondary antibodies were added according to the instructions and DAPI (Thermo Fisher, USA) was applied to stain the cell nuclei. Finally protein expression level was visualized and images were captured by using Olympus FV3000 confocal microscope.

### Human-mouse tumor xenograft model and animal imaging

Animal studies were conducted with the approval of Ethics Committee of the Laboratory Animal Center of Wuhan University of Science and Technology. 4-weekold male BALB/c nude mice were purchased from Beijing Huafukang Experimental Animal Co, Ltd. and maintain in specific pathogen free condition. 1 × 10^7^ cells of CENPA knockdown A549 cells and A549 cells in control were injected simultaneously into different groups of mice. After 18 days of incubation, mice were used for subsequent different experiments.

For imaging, mice were first anesthetized with isoflurane. Meanwhile mice were injected intraperitoneally with D-Luciferin at a dose of 150 mg/KG. Bioluminescence imaging signals were measured 10 minutes after D-Luciferin injection, and were carried out by placing the region of interest (ROI) on the mouse. The light intensity (in photons /s^−1^ /mm^−2^) is measured within this ROI.

### Screening for differentially expressed genes (DEG)

Lung adenocarcinoma mRNA sequencing profiles were from the TCGA database, and the R packages limma, heatmap and ggplot2 were applied to process the downloaded data and present the top 50 differentially expressed genes in the form of heatmap and volcano plot [11, 12]. The criteria for determining differential gene expression were *p* < 0.05 and absolute value of logFC >2.

### WGCNA identifies key modules and hub genes

Based on the results of the DEG screen, we applied the WGCNA and the cancer stemness index (mRNAsi) to obtain modules with different correlations to this index [5]. After determining the module to be studied, we screened out the key genes in this module according to the gene significance (GS) and module membership (MM) values. Then we used the software Cytoscape to determine the hub gene. The CytoHubba plug-in can detect and lock the most related genes among these key genes by using the maximal clique centrality (MCC) and label these ten genes with different colors to represent different degrees of relatedness [13, 14]. Depending on their degree of correlation with the index in LUAD, red represents high correlation, orange represents moderate correlation and yellow represents low correlation.

### Functional enrichment analysis

GO analyses were utilized to characterize the function of DEGs. And these researches were carried out by applying dose, and ggplot2 R packages [12, 15]. *P* < 0.05 and *FDR* <0.05 were used as the judgement criteria.

### Construction of risk score models

The R packages glmnet and survival were used to complete the LASSO (least absolute shrinkage and selection operator) regression analysis and to select genes for the model [16, 17]. Key genes identified on the basis of MM and GS values were first screened for prognosis value by univariate Cox regression analysis. Selected genes were then subjected to LASSO regression analysis to calculate their prognostic value and variable coefficients. We then utilized the following computational formula to obtain the risk score.


$$\text{risk score}=\sum_{i=1}^{n}\text{Coefi}\times \text{Xi}$$

Coef is the coefficient of the gene and X represents the expression level of the gene. The mean of the risk scores was used as a criterion to classify the samples into high and low risk groups. Kaplan-Meier survival analysis was used to analysis differences in survival between the two groups. Univariate and multivariate Cox analyses were used to analysis whether risk score and clinical characteristics could be independent prognostic factors.

### Gene set enrichment analysis

The c2.cp.kegg.v7.4.gene sets and Hallmark collections were obtained from Molecular Signatures Database (MSigDB) and subsequently analyzed by applying GSEA software (version: 4.0.3) to analyze the data. The c2.cp.kegg.v7.4.gene sets was used as the reference gene set. [18, 19].

### Statistical analysis

All experimental data were processed by the software GraphPad Prism 8 and were presented as mean ± standard deviation. An unpaired *t*-test was used to compare the differences between the two groups. The *p-*value less than 0.05 was considered to be significantly different (^*^P < 0.05; ^**^P < 0.01; ^***^P < 0.001; ^****^P < 0.0001).

### Ethics approval and consent to participate

All mouse experimental procedures were evaluated and authorized in strict accordance with the guiding principles of the Animal Protection and Use Committee of Wuhan University of Science and Technology and in accordance with the “Hubei Province Experimental Animal Management Regulations.”

### Consent for publication

All authors have read this manuscript and approved for submission.

### Availability of supporting data

The data generated during this study are included in this article and its supplementary information files are available from the corresponding author on reasonable request.

## RESULTS

### Screening for differentially expressed genes and differences in mRNAsi

**Figure 1:**
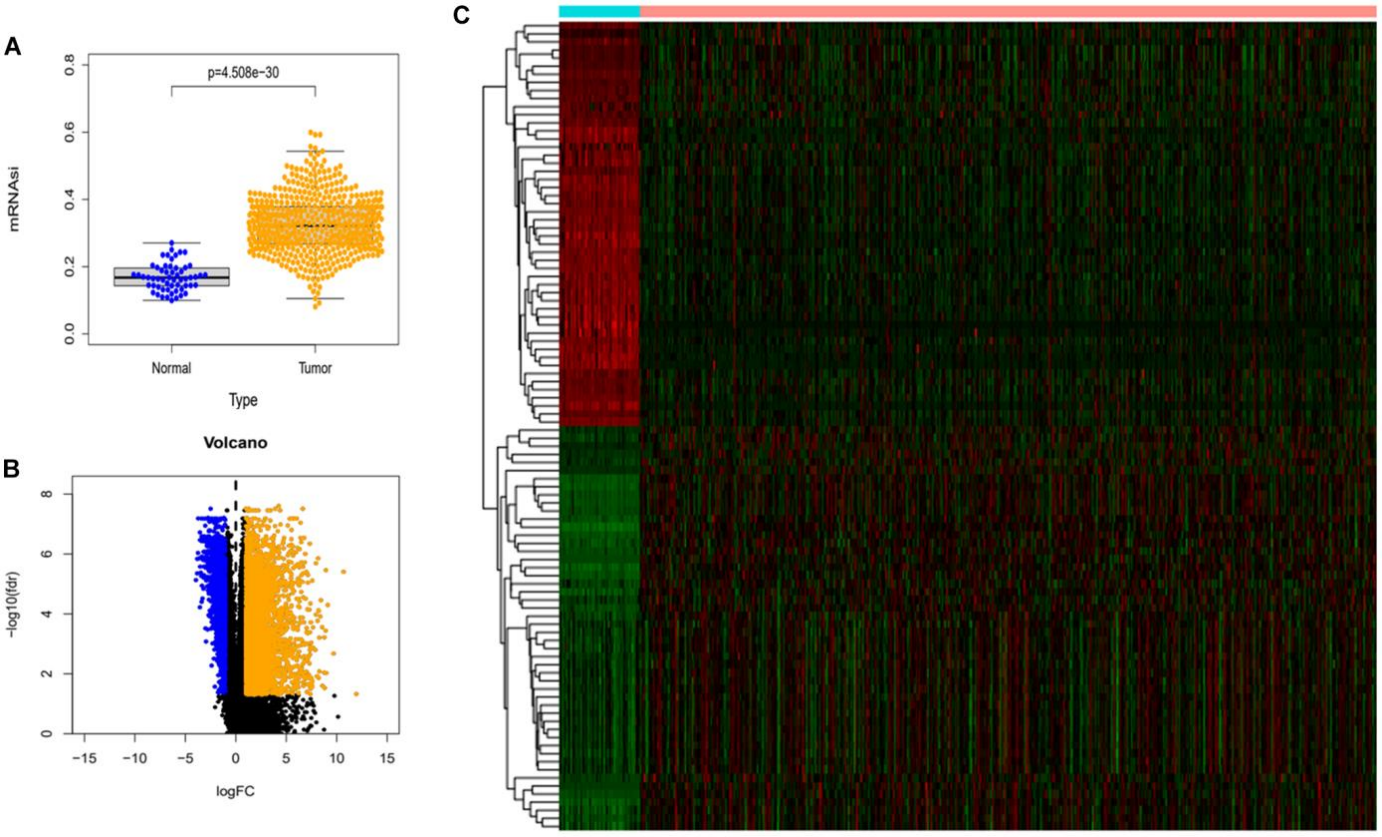
**Differences in mRNAsi and sample gene expression.** (**A**) Differences in mRNAsi between normal and tumor tissues in lung adenocarcinoma. (**B**) Volcano map of differentially expressed genes. Green dots represent genes that are down-regulated, red dots represent genes that are up-regulated, and black dots represent no significant change. (**C**) The top 50 differentially expressed genes in LUAD cancer disease presented as a gene expression heat map. *P* < 0.05.

We downloaded mRNA expression profile data for lung adenocarcinoma from the TCGA database as well as clinical data for the samples. As shown in Figure 1A, there was a significant difference in mRNAsi levels between these two groups. The mRNAsi in the tumor group was much higher than that in the normal samples. Finally, we screened the expression profile data for differentially expressed genes (DEGs) with limma R package. In total, we found 6778 differentially expressed genes, of which 5178 were up-regulated and 1600 were down-regulated. We extracted the top 50 expression data of the DEG and plotted them as heat maps (Figure 1B and 1C).

### Construction of the WGCNA gene co-expression network

**Figure 2:**
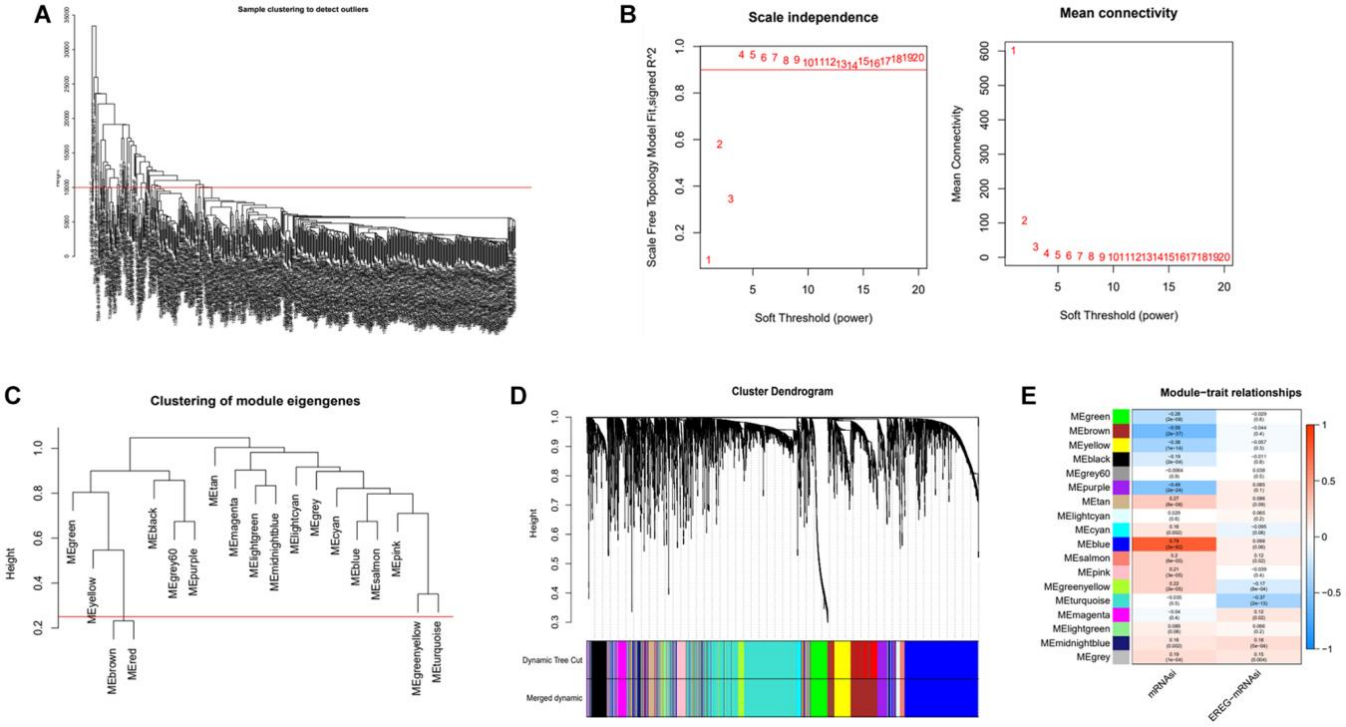
**Identification of cancer stemness index-related modules by WGCNA.** (**A**) Samples above the red line were removed because they were considered as the deflection of gene expression. (**B**) This represents the correlation coefficient R^2^ and mean connectivity in the scale-free network. (**C**) Calculate similarity between modules and merge modules with high similarity. (**D**) Hierarchical clustering of gene modules. (**E**) Heatmap of the correlation ship between gene modules and cancer stemness index.

Based on DEGs and mRNAsi values, we applied the WGCNA algorithm to build a gene co-expression network to screen for cancer stemness index-related modules. Firstly, samples with the deflection of gene expression were deleted (Figure 2A). Then considering both the scale-free correlation coefficient and the average module connectivity, we selected 4 as the soft threshold parameter (Figure 2B). Thirdly, we calculate the similarity of modules and merge modules that have a high degree of similarity (Figure 2C and 2D). A total of 18 co-expression modules are established and named in different colors for the convenience of description (Figure 2E). As shown in 2E, the blue (R^2^ = 0.79. *p*.value = 2e-82) and turquoise (R^2^ = −0.37. *p*.value = 2e-13) modules were highly correlated with mRNAsi. Because of its maximum positive correlation, we chose the blue module as the follow-up study target.

### Identification of key genes and their functional enrichment and correlation analysis

**Figure 3:**
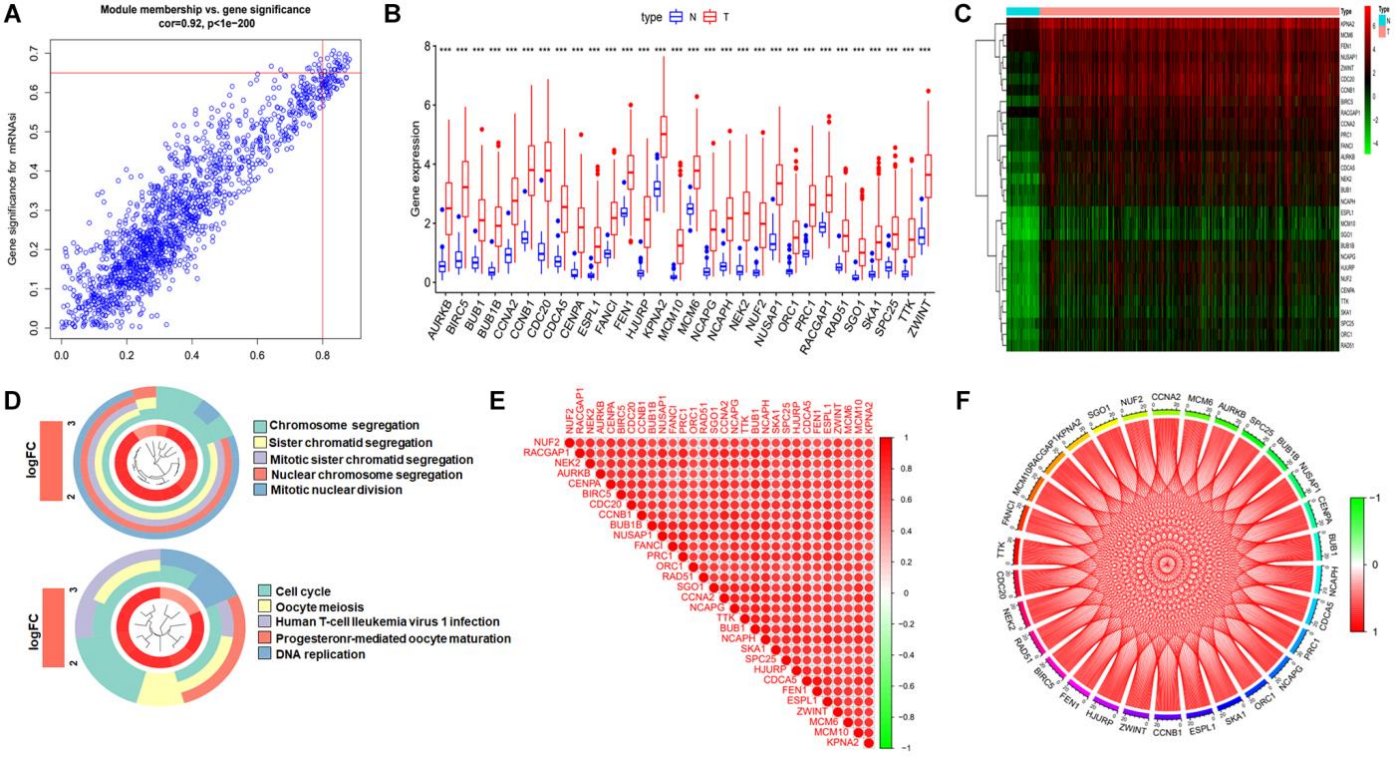
**Identification the key genes and expression, functional enrichment and correlation analysis of these genes.** (**A**) Scatter plot of maximum positive correlation with cancer stemness index (mRNAsi). (**B** and **C**) Box plot of the difference in expression of key genes between tumor and normal tissue. (**D**) Functional enrichment analysis of key genes. (**E** and **F**) Analysis of the correlation of key genes at the transcriptional level, red represents for positive correlation and green represents for negative correlation.

The blue module is the most positively correlated with the mRNAsi index. Model membership (MM) values represent the magnitude of the relationship between the gene and this module, and gene significance (GS) values represent the association of the gene with mRNAsi. To further narrow down the study, we used MM > 0.8 and GS >0.65 as criteria to select key genes (Figure 3A). In total, there were 1554 genes in the blue module, and we finally identified 30 key genes. We then extracted the expression profile of these genes and presented the differences in expression between tumor and normal tissues in the form of heatmap and box plots (Figure 3B and 3C). To investigate the functions of these key genes, we performed GO functional enrichment analysis, which revealed that these genes are mainly responsible for chromosome segregation, DNA replication, and microtubule movement, all of which are highly relevant to the cell cycle (Figure 3D). We also performed correlation analysis of key genes to demonstrate the high correlation of these genes within the consent module as shown in the Figure 3E and 3F.

### Identification of hub genes by protein-protein Interaction (PPI) networks and validation their expression

**Figure 4:**
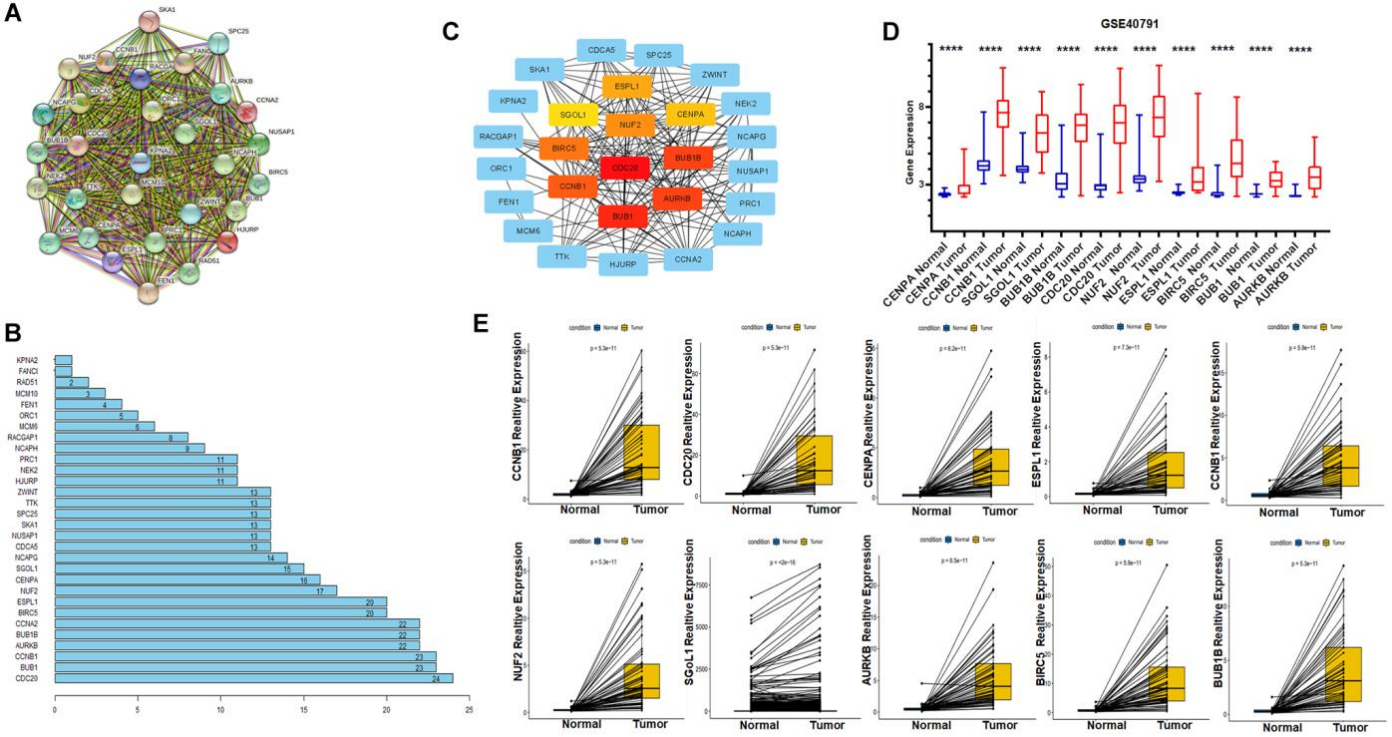
**Identification of hub genes using Protein-Protein Interaction Network and Cytoscape; GEO dataset validates expression of key genes.** (**A**) Protein- Protein Interaction Network of Key Genes. (**B**) Number of edges per key gene. (**C**) The CytoHubba plug-in identifies hub genes and marks them with different colours, red for high correlation, orange for moderate correlation and yellow for low correlation. (**D**) External validation of the GSE40791 dataset for differential expression of hub genes in tumor and normal tissues. (**E**) Paired expression analysis of hub genes from TCGA.

To better understand the interactions of these genes, we build a protein-protein interaction network through the online website STRING (https://cn.string-db.org/) (Figure 4A). In this network, there are 30 nodes and 413 edges, and the PPI enrichment *p*-value: <1.0 × 10^−16^. In addition, we show the number of connections between nodes as a bar diagram (Figure 4B). To further narrow down the study, we applied Cytoscape software to screen and visualize the hub genes. Ten hub genes were identified through the Maximal Clique Centrality (MCC) value. They are SGOL1, CENPA, ESPL1, NUF2, BIRC5, CCNB1, BUB1B, AURKB, BUB1, CDC20 and are labelled with different colours (Figure 4C). Finally, to demonstrate the different expression level of ten hub genes in tumor and normal tissues, we firstly validated it with the GSE40791 dataset downloaded from the GEO data base and subsequently also performed a paired analysis of the expression data of TCGA database. The results showed significant differences in the expression of these genes in normal versus tumor tissue (Figure 4D and 4E).

### Establishing a risk assessment model

**Figure 5:**
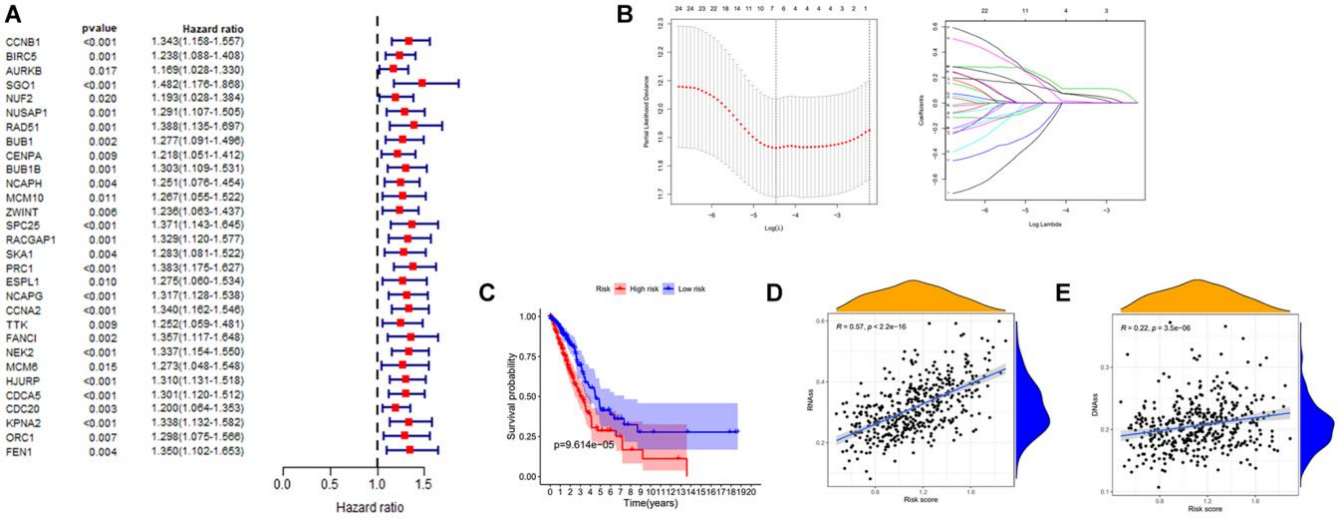
**Establishment of risk prognostic model.** (**A**) Univariate Cox analysis of the prognostic value of key genes. (**B**) LASSO Cox regression analysis of key genes. (**C**) The patient samples were divided into high and low risk groups based on risk score and the OS of the groups were analyzed using Kaplan-Meier. Red represents the high risk group and blue represents the low risk group. (**D** and **E**) Analysis of the linear relationship between risk score and cancer stemness index. Abbreviations: LASSO: least absolute shrinkage and selection operator; OS: overall survival.

To investigate the prognostic value of these key genes in lung adenocarcinoma, we first applied univariate Cox regression analysis to perform a preliminary screening of the prognostic role of these genes. As shown in the Figure 5A and Table 1, all of these key genes are of prognostic significance and for further clarification of the scope of the study. We loaded these genes into the LASSO regression analysis model to build a risk assessment model (Figure 5B). As a result, a 6-gene model (CCNB1, CCNA2, TTK, CENPA, PRC1, NEK2) was screened out. Four of these six genes were positive correlated with overall survival, and two were negative correlated. We obtained the risk score of LUAD patients with the corresponding coefficients. The calculation method is as follows: (expression level of TTK × −0.134+ expression level of NEK2 × 0.114+ expression level of CCNB1 × 0.116+ expression level of CCNA2 × 0.194+ expression level of CENPA × −0.179+ expression level of PRC1 × 0.16). Samples were classified into high and low-risk groups based on the mean value of risk score. There is a significant difference in survival rate between the two groups of samples (Figure 5C). In the end, we analyzed the linear relationship of risk score and cancer stemness index. As shown in Figure 5D and 5E, risk score increases with cancer stemness index. This would indicate that the risk score is positively correlated with the cancer stemness index, which can also be used to assess the amount of tumor stemness in a tumor.

**Table 1:** Results of the key genes in the Univariate Cox regression analysis.

**ID**	**Univariate Cox analysis**			
	**HR**	**HR.95L**	**HR.95H**	***P* value**
CCNB1	1.342789	1.158325	1.556631	**9.26E-05**
BIRC5	1.237655	1.087878	1.408052	**0.001196**
AURKB	1.169464	1.028468	1.329789	**0.016931**
SGO1	1.48225	1.175871	1.868456	**0.000864**
NUF2	1.192925	1.028004	1.384302	**0.020137**
NUSAP1	1.290575	1.106949	1.50466	**0.001124**
RAD51	1.388178	1.135332	1.697335	**0.001388**
BUB1	1.277222	1.090771	1.495544	**0.002373**
CENPA	1.217873	1.05066	1.411697	**0.008902**
BUB1B	1.302922	1.108937	1.530841	**0.001295**
NCAPH	1.25077	1.075752	1.454262	**0.003621**
MCM10	1.267074	1.055102	1.521632	**0.01127**
ZWINT	1.236082	1.063324	1.436908	**0.005792**
SPC25	1.370872	1.142546	1.644827	**0.00069**
RACGAP1	1.328666	1.119641	1.576713	**0.001138**
SKA1	1.282937	1.081111	1.522442	**0.004331**
PRC1	1.382863	1.175185	1.627241	**9.45E-05**
ESPL1	1.275134	1.059703	1.534361	**0.01005**
NCAPG	1.317074	1.127774	1.538148	**0.000504**
CCNA2	1.340231	1.162207	1.545524	**5.64E-05**
TTK	1.252381	1.058702	1.481491	**0.008653**
FANCI	1.356766	1.117141	1.647791	**0.00209**
NEK2	1.337364	1.153986	1.549882	**0.000112**
MCM6	1.273415	1.047534	1.548002	**0.015262**
HJURP	1.310447	1.131353	1.517893	**0.000311**
CDCA5	1.301448	1.120259	1.511943	**0.000572**
CDC20	1.200084	1.064279	1.353219	**0.002914**
KPNA2	1.338003	1.13198	1.581524	**0.000642**
ORC1	1.297608	1.075372	1.565772	**0.006566**
FEN1	1.349571	1.101617	1.653334	**0.0038**

Bold words mean *P* value is less than 0.05; Abbreviations: HR: hazard ratio; L: low; H: high.

**Figure 6:**
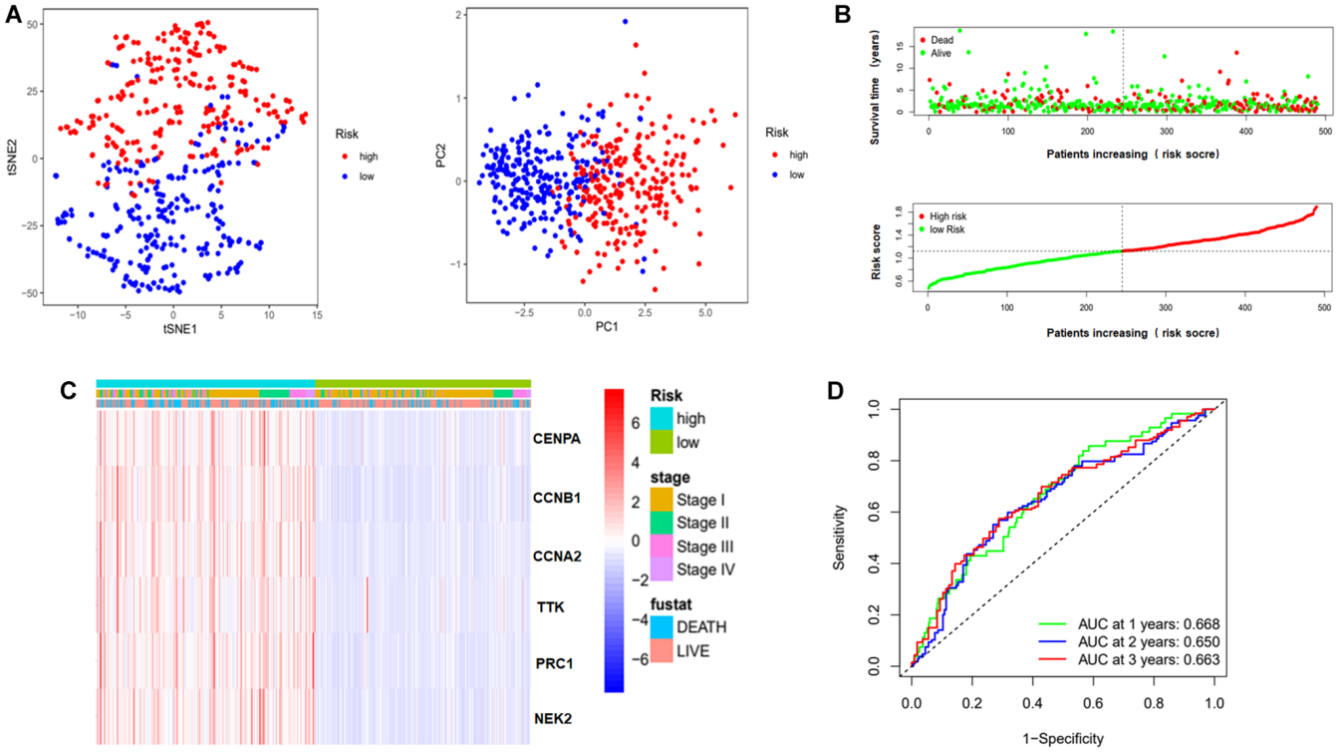
**Evaluation of risk model.** (**A**) PCA and t-SNE analysis to assess the sample of the risk model. Red dots represent high risk group, blue dots represent low risk group. (**B**) Risk score maps and survival status maps of patients in the high and low risk groups. In the survival status map, red dots represent death, green dots represent survival. In the risk score maps, green represents low risk and red represents high risk. (**C**) Clinical heat map representing the relationship between risk genes and clinical characteristics. (**D**) ROC curves to evaluate the prognostic effect of this model on overall survival at 1, 2, 3 years. Abbreviations: t-SNE: t-distributed stochastic neighbor embedding; PCA: Principal Component Analysis; AUC: area under the curve; ROC: receiver operating characteristic curve.

We also evaluated this model through Principal Component Analysis (PCA) and t-Distributed Stochastic Neighbor Embedding (t-SNE) analysis. Figure 6A shows that the high-risk and low-risk samples were well separated based on risk score. Figure 6B shows an increase in the proportion of patient deaths in the high-risk group. And the clinical heat map shows no differences in risk score in some clinical characteristics, including pathological stage and fustat (Figure 6C). Finally, ROC curve was utilized to judge the accuracy of this risk model for survival rate. The results showed that the AUC values at 1, 2 and 3 years were 0.668, 0.650 and 0.663 respectively (Figure 6D). This indicates that this prognostic model has a high accuracy.

### Evaluate the value of this risk model for clinical application

**Figure 7:**
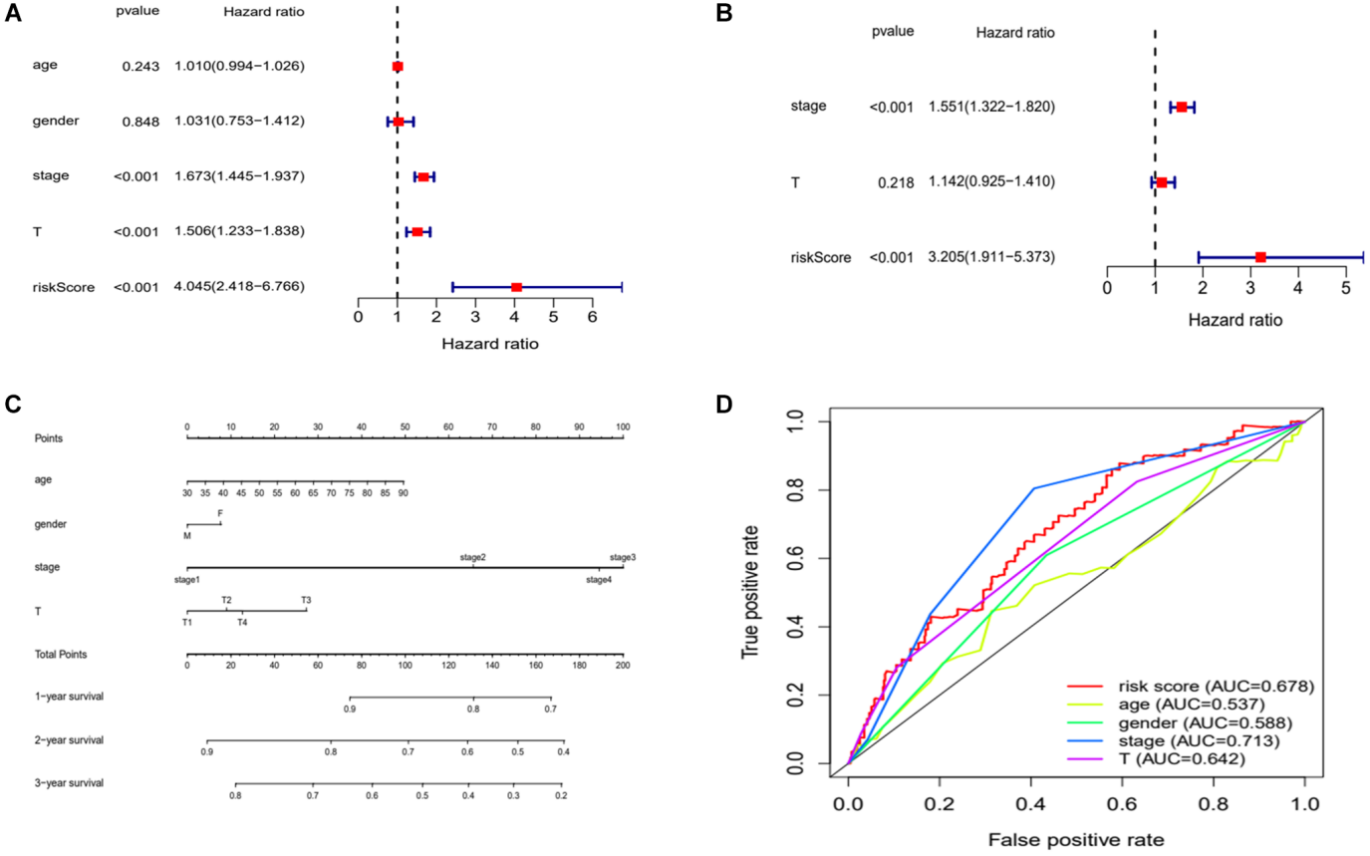
**Evaluation of risk model.** (**A** and **B**) Univariate and multivariate Cox analysis of risk scores and clinical characteristics. (**C**) A nomogram with clinical characteristics predicts 1,2,3 years OS of lung adenocarcinoma patients. (**D**) ROC curves for clinical characteristic. Abbreviations: AUC: area under the curve; ROC: receiver operating characteristic curve.

We sought to assess whether clinical characteristics corresponding to the risk score could also be used as independent risk factors to influence the prognosis of patients. We applied regression analyses calculated the risk score and their corresponding clinical characteristics. We obtained the following results. The univariate Cox regression analyses found that pathological stage, tumor size and risk score significantly affected the prognosis of LUAD patients (Figure 7A). Further, multivariate Cox regression analyses found that tumor size and risk score could significantly influence prognosis as independent risk factors (Figure 7B). A prognostic nomogram clinicopathological parameters was also established (Figure 7C). We then applied ROC curves to verify these results and the AUC values for the different clinical characteristics were risk score (0.678), gender (0.588), age (0.537), pathological stage (0.713), and tumor size (0.642) (Figure 7D). Considering the above results together, pathological staging can be also used as an independent risk factor for prognosis (Table 2).

**Table 2:** Results of the risk score and clinical characteristics in the Univariate and Multivariate Cox regression analysis.

**Parameter**	**Univariate analysis**				**Multivariate analysis**			
	**HR**	**HR.95L**	**HR.95H**	***P* value**	**HR**	**HR.95L**	**HR.95H**	***P* value**
age	1.009622	0.993506	1.026	0.243439				
gender	1.031244	0.753098	1.412119	0.847868				
stage	1.672796	1.444746	1.936843	**5.97E-12**	1.551218	1.322385	1.81965	**6.99E-08**
T	1.505592	1.233059	1.838361	**5.91E-05**	1.141702	0.924765	1.409528	0.217757
Risk Score	4.045005	2.418103	6.766488	**1.02E-07**	3.204635	1.911201	5.373421	**1.00E-05**

Bold words mean *P* value is less than 0.05; Abbreviations: HR: hazard ratio; L: low; H: high.

### Validating the prognosis and expression of the model risk genes

**Figure 8:**
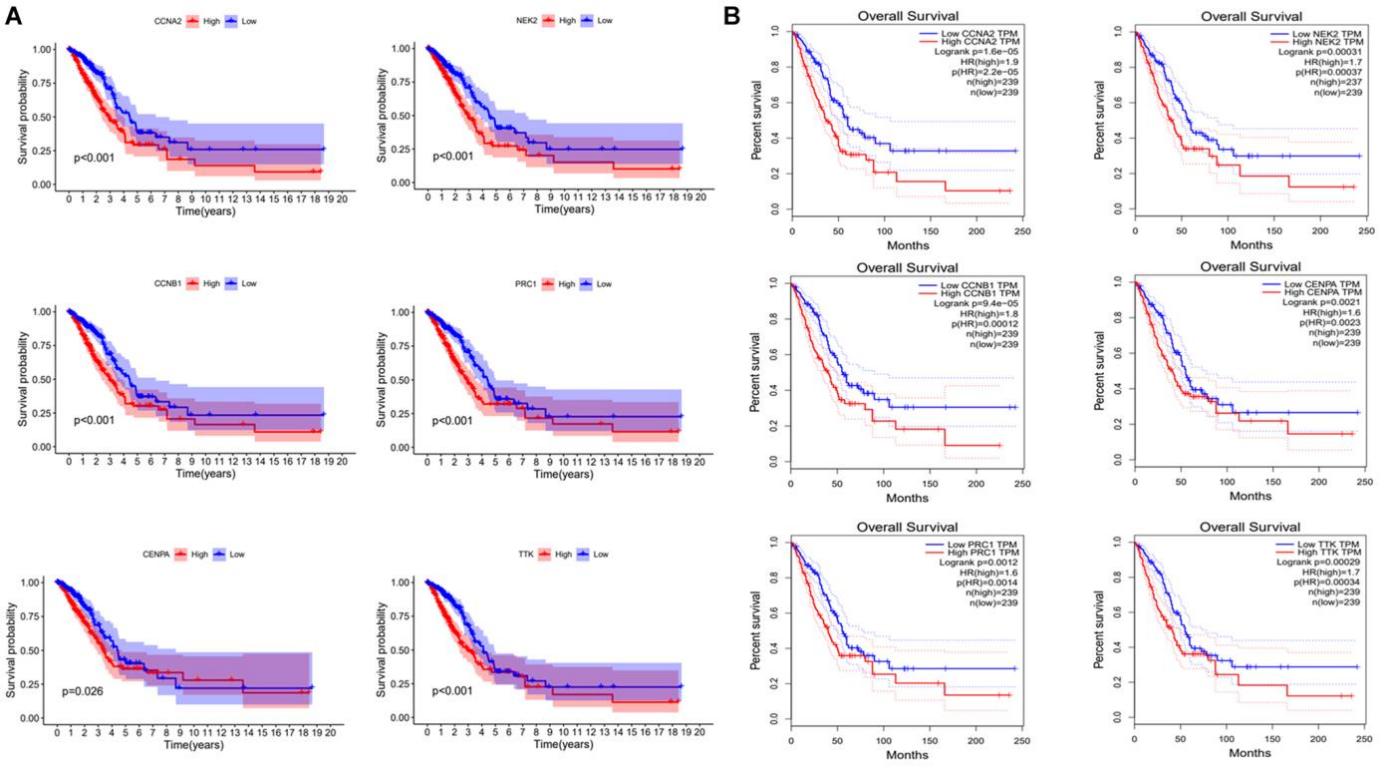
**Validating the prognostic value of risk genes.** (**A**) Combining TCGA clinical data and expression data to analyze OS of risk genes. (**B**) OS analysis of risk genes by using the GEPIA website.

To verify the prognostic role of these genes, we calculated the survival curves of these genes (Figure 8A). The *p*-values of these curves were less than 0.05. Meanwhile, we also utilized other online databases GEPIA (http://gepia.cancer-pku.cn/detail.php?gene=DLGAP1-AS1) to verify the prognostic role of these genes and obtained similar results (Figure 8B). In summary, the results suggest that these genes have a good prognostic potential in lung adenocarcinoma.

**Figure 9:**
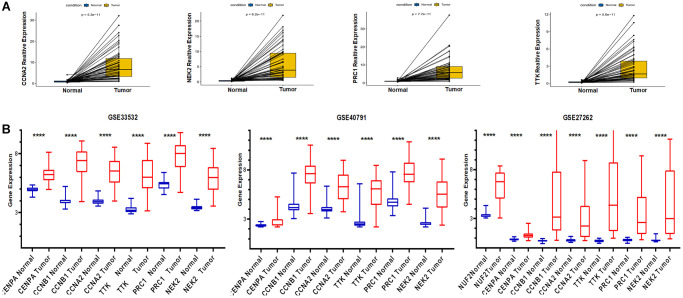
**Application of TCGA and GEO databases to validate the expression of risk genes.** (**A**) Pairwise analysis from TCGA. (**B**) GEO database external validation of risk genes expression.

Validation of mRNA expression levels of risk genes was performed using both TCGA pairwise analysis and GEO external verification. We first performed pairwise analysis of these six genes using the R package limma and ggpubr in the expression profile downloaded from TCGA. The pairwise analysis of CCNB1 and CENPA has been shown in Figure 4E. The remaining results were as shown in Figure 9A. Risk genes were abnormally high expressed in tumor tissue. We have also validated the expression of these genes by means of microarray. The GSE33532, GSE40791, GSE27262S datasets were used for external validation. As shown in Figure 9B, these risk genes were highly expressed in all three datasets, providing external validation of the expression.

### Multi-gene set enrichment analysis

**Figure 10:**
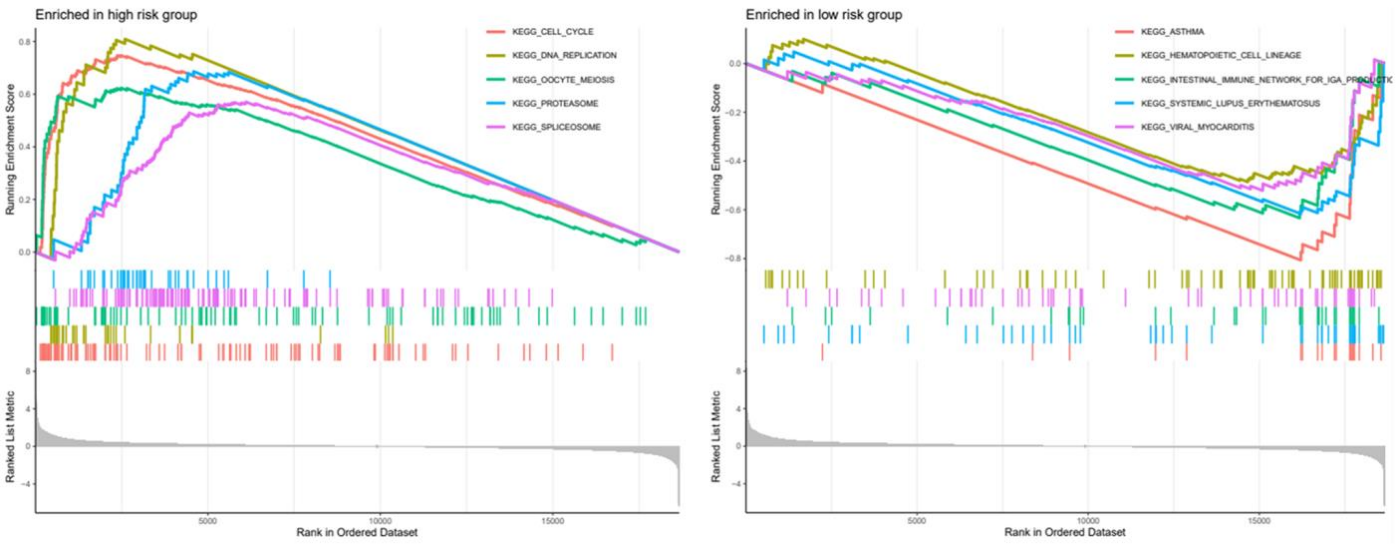
GSEA analysis of high and low risk groups and presentation of the top five analysis results.

To more understand the role of this six-gene model in lung adenocarcinoma. We performed GSEA analysis of these six genes in different risk groups separately and show the functional enrichment results for the top 5 in the Figure 10. In the high-risk group these genes are mainly responsible for cell cycle, DNA replication, oocyte meiosis, spliceosome, proteasome, etc. In the low-risk group these genes are mainly associated with asthma, hematopoietic cell lineage, etc.

### CENPA possesses the ability to regulate the properties of tumor stem cells in LUAD

**Figure 11:**
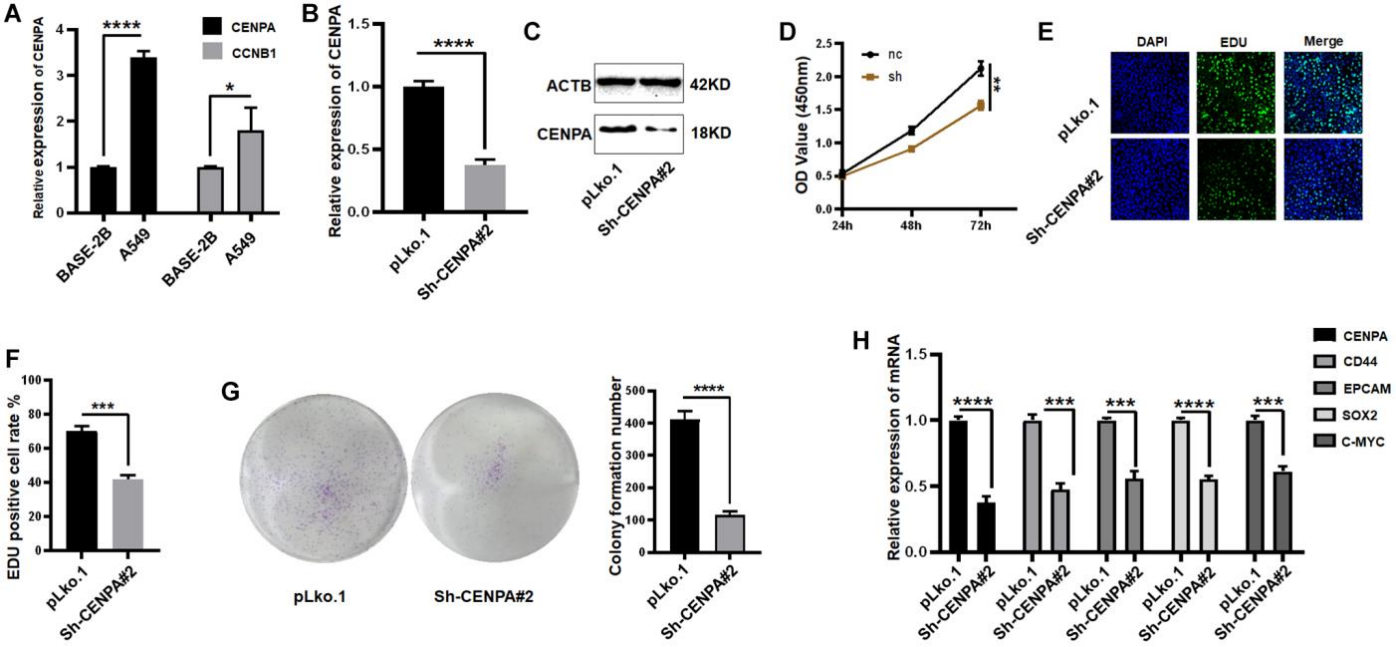
**Identification of a risk prognostic gene regulating tumor stemness in lung adenocarcinoma cells *in vitro*.** (**A**) Application of QPCR to compare CENPA and CCNB1 mRNA expression in tumor cells and normal epithelial cells. (**B** and **C**) QPCR and western blot to validate the effect of sh-RNA knockdown. (**D**–**G**) The effect of knocking down CENPA on cell proliferation ability was examined by CCK-8, EDU and clone formation respectively. (**H**) QPCR detection of tumor stem cell biomarkers. (^*^P < 0.05; ^**^P < 0.01; ^***^P < 0.001; ^****^P < 0.0001).

To further explore the biological functions and regulatory mechanisms of these genes in lung adenocarcinoma. We first selected the intersection of hub genes and risk model genes, and the result were CCNB1 and CENPA (Supplementary Figure 1). We then applied QPCR to screen the expression of both in the lung normal epithelial cell line BSAE-2B and the lung adenocarcinoma cell line A549, and finally identified CENPA as the final study target. As shown in Figure 11A, the expression of CENPA was approximately 3.4-fold higher in A549 than in BASE-2B and approximately 1.8-fold higher in CCNB1.

A stable cell line was constructed by knocking down CENPA in the A549 cell by means of stable expression of small hairpin RNA. The knockdown efficiency of small hairpin RNA was approximately 65% (Figure 11B and 11C). CCK-8 assay was utilized to test the impact of CENPA knockdown on the proliferative capacity of the cells. The results showed that knockdown of CENPA caused ~26% reduction in proliferative capacity (Figure 11D). Subsequent EDU and colony formation assays also yielded similar results (Figure 11E–11G). We then equally explored the effect of knockdown CENPA on the maintenance of tumor stemness.

**Figure 12:**
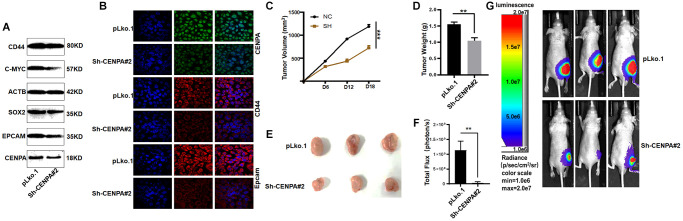
**Validating the effect of CENPA on tumors *in vivo*.** (**A** and **B**) Validation of protein expression levels of cancer stem cell biomarkers by western blot and immunofluorescence. (**C**) Tumor volume curve of control group and knockdown group. (**D**) Tumor weights in control and knockdown groups. (**E**) Image of xenograft tumors in different groups of mice. (**F** and **G**) Animal imaging technology to detect differences between control and knockdown groups. (^*^P < 0.05; ^**^P < 0.01; ^***^P < 0.001; ^****^P < 0.0001).

First, we used QPCR to detect several commonly used biomarkers of cancer stem cells, which are CD44, EpCAM, SOX2, C-MYC. As shown in Figure 11H, when CENPA was knocked down, the levels of these cancer stem cell biomarkers were significantly decreased. Then we utilized western blot and immunofluorescence techniques to the detect protein level expression of these biomarkers (Figure 12A and 12B). The results were consistent with the mRNA levels. The knockdown of CENPA would affect the expression of these indicators.

To more accurately evaluate the effect of CENPA on tumors *in vivo*, CENPA-knockdown stably transfected cell lines and control cells were injected subcutaneously into immunodeficient nude mice at the same time. The object of this experiment is to test whether CENPA affects tumor growth *in vivo*. After 18 days of *in vivo* incubation, we found a significant decrease in tumor volume and weight in the knockdown group compared to the control group. The average decrease in tumor weight in the experimental group was 0.5 grams and average decrease in tumor volume was 460 cubic millimetres (Figure 12C–12E). The results obtained from the animal imaging technique were similar to those described above, with the control group showing a significantly higher fluorescence signal than the test (Figure 12F and 12G). Such results suggest that CENPA expression is strongly associated with tumorigenesis and progression.

## DISCUSSION

Tumor stem cells play an integral role in the development, progression, metastasis, invasion, drug resistance and recurrence of various tumors [7, 20]. It is therefore particularly significant to identify genes that are highly associated with tumor stem cell properties and to investigate the mechanisms by which these genes regulate tumor stemness. Malta et al. used the innovative one-class logistic regression machine-learning (OCLR) algorithm in combination with public data from TCGA database to obtain two cancer stemness indices, one (mRNAsi) for gene expression assessment and the other (mDNAsi) for gene epigenetic modification assessment. This parameter has been used to identify tumor stem cell-associated genes in a variety of cancers, such as endometrial cancer, gastric cancer, pancreatic cancer, etc. We then screened for differentially expressed genes in preparation for subsequent studies.

We correlated mRNAsi with gene expression through WGCNA. By the correlation coefficients of the different modules, we selected the module with the largest positive association with mRNAsi. We then extracted the expression data of the genes in this module and performed functional enrichment analysis. The results showed that these key genes are mainly responsible for and involved in cell cycle, chromosome segregation, centrosome assignment, etc. These results are consistent with the current view that tumor stem cells possess a strong capacity for self-renewal [21]. By using the Protein-Protein Interaction Networks and the software Cytoscape, we screened ten hub genes, of which CDC20 is marked in red for high correlation. Previous findings have discovered that CDC20 plays a significant function in the maintenance of tumor stem cell properties. Knockdown of CDC20 inhibited the expression of stem cell properties, self-renewal capacity, chemoresistance, invasiveness and tumorigenicity-related genes in prostate CSCs. In addition, CDC20 was able to promote the degradation of core members of the Axin1 and β-linked protein disruption complexes, followed by reduced phosphorylation of β-linked proteins, thereby promoting the entry of β-linked proteins into the nucleus to enhance the self-renewal capacity of CD44^+^ prostate CSCs [22].

The mRNAsi-based risk model has been applied to many cancers. According to this, we created a six-gene risk model. And the results of the Kaplan-Meier analysis showed that patients in the high-risk group possess a poor prognosis. Risk score and clinicopathological stage could be used as independent prognostic factors after univariate and multivariate Cox regression analysis. This suggests that this risk model can be used in conjunction with clinicopathological stage to determine a patient’s prognostic risk, allowing for individual precision in the treatment and management of patients.

All of the above risk-associated genes are involved in the cell cycle .Never in mitosis gene A-related kinase 2 (NEK2) is a cell cycle-regulating serine-threonine protein kinase, and several reports have focused on its role in chromosome instability, tumorigenesis and resistance to chemotherapy [23–25]. It has been found to be abnormal expressed in various tumors such as colon, prostate and pancreas [26–30]. Serine threonine protein kinase (TTK), also known as monopolar spindle1, is an indispensable component of the spindle assembly checkpoint, and is overexpressed in various tumors and also plays a momentous function in the development and maintaining tumor stem cells [31]. Its main function is responsible for chromosome segregation and DNA damage repair [32]. In triple negative breast cancer, overexpression of TTK is associated with tumor progression and prognosis, and its knockdown inhibits cancer cell invasion and proliferation [33, 34]. Protein Regulator of Cytokinesis 1(PRC1) is a microtubule-associated protein. In ovarian and cholangiocarcinoma, patient with abnormal expression of PRC1 had poor prognosis [35, 36]. In this study, our findings were consistent with previous studies in which PRC1 was highly expressed in lung adenocarcinoma and correlated with patient prognosis [37]. CCNB1 and CCNA2 are up-regulated in a variety of tumors and promote tumor proliferation, and also have a prognostic role [38, 39]. In ovarian, hepatocellular and prostate cancers, CENPA functions as a promoter of tumor growth, proliferation and migration [40–42]. In this study, we also found that patients who are high in CENPA expression in lung adenocarcinoma hold a poor prognosis, which is consistent with previous findings [43]. Subsequently, CENPA was found to own the ability to regulate tumor stemness and proliferation in *in vivo* and *in vitro* studies However, our study appears to be the first to suggest that CENPA not only regulates tumor stemness but also has an independent prognostic effect in lung adenocarcinoma. This provides a new direction and target for follow-up research and treatment.

In summary, we have analyzed mRNAsi-related genes in lung adenocarcinoma and developed a risk model. This provides a new way to study the stem cell mechanism of lung adenocarcinoma and the prognosis of patients. In addition, it has been experimentally verified that CENPA has the ability to regulate tumor stemness. However, some limitations of our study still exist. Firstly, this study was unable to validate the risk model using external data sets because no clinical information was available for lung adenocarcinoma in databases such as GEO and ICGC. Second, the mechanisms by which CENPA regulates tumor cell stemness need to be investigated in more detail. In future studies, we will apply more comprehensive clinical information and data sets for validation and will design more detailed *in vivo* and *in vitro* experiments to verify its regulatory mechanisms.

## Supplementary Materials

Supplementary Figure 1
