# Supplementary figures and images for: CENPA regulates tumor stemness in lung adenocarcinoma
Source: Aging (Albany NY). 2022 Jul 11;14(13):5537–53. doi: 10.18632/aging.204167 (PMC9320546; doi:10.18632/aging.204167)

SUPPLEMENTARY FIGURE

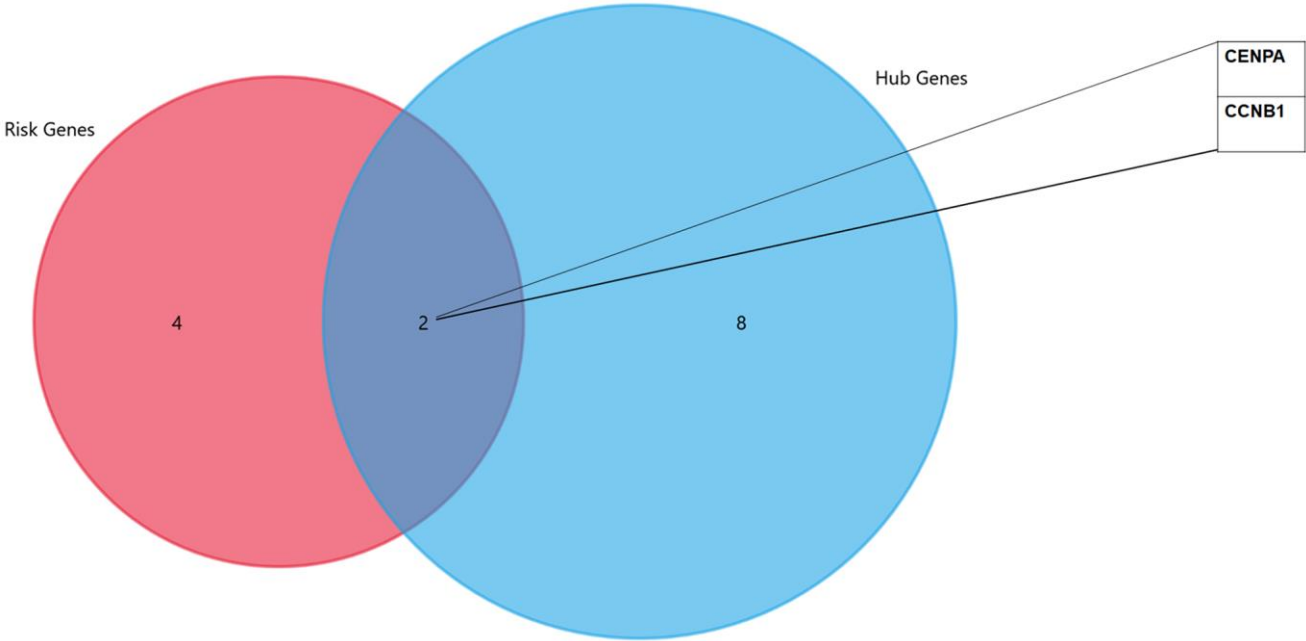

Supplementary Figure 1. The intersection of risk genes and hub genes.

Supplement: Supplementary Figure 1 [file aging-14-204167-s001.pdf]
